# Molecular glue degrader for tumor treatment

**DOI:** 10.3389/fonc.2024.1512666

**Published:** 2024-12-12

**Authors:** Yuhan Hu, Yan Yan, Jiehao Wang, Jiangxue Hou, Quande Lin

**Affiliations:** ^1^ Department of Hematology, Affiliated Cancer Hospital of Zhengzhou University & Henan Cancer Hospital, Zhengzhou, China; ^2^ Department of Infectious Diseases, Zhoukou Central Hospital, Zhoukou, China; ^3^ Department of Gastroenterology, Zhengzhou First People's Hospital, Zhengzhou, China

**Keywords:** molecular glue degrader, tumor, design strategies, mechanism, clinical trials

## Abstract

Targeted Protein Degradation (TPD) represented by Proteolysis-Targeting Chimeras (PROTAC) is the frontier field in the research and development of antitumor therapy, in which oral drug HP518 Receives FDA Proceed Authorization for its IND Application for Prostate Cancer Treatment. Recently, molecular glue, functioning via degradation of the target protein is emerging as a promising modality for the development of therapeutic agents, while exhibits greater advantages over PROTAC, including improved efficiency, resistance-free properties, and the capacity to selectively target “undruggable” proteins. This marks a revolutionary advancement in the landscape of small molecule drugs. Given that molecular glue research is still in its early stage, we summarized the mechanisms of molecular glue, the promising drugs in clinical trials and diverse feasible design strategies for molecular glue therapeutics.

## 1 Introduction

Molecular glue emerges as a class of diminutive compounds orchestrating the proximity necessary for the meticulous regulation of specific biological phenomena, including signal transduction, transcription, chromatin modulation, as well as protein folding, localization, and degradation (1). Operating as chemical facilitators, molecular glue effectively promotes the dimerization or co-localization of two proteins by instigating ternary complexes, thereby engendering diverse biological and pharmacological functions (2–4). The story of molecular glue degraders begins with thalidomide. Thalidomide began as a drug to treat pregnancy sickness, but was shelved in the early 1960s due to horrific birth defects (5). It was approved for the treatment of leprosy in 1998 and for multiple myeloma in 2006 (6, 7), but its mechanism remains unclear. In 2010, Hiroshi Handa’s team discovered that thalidomide binds the E3 ubiquitin ligase CRBN(cereblon), while Handa first hypothesized that the drug inhibited ubiquitination (8, 9). In 2014, research group revealed that lenalidomide, a thalidomide derivative, is a glue that enables ubiquitination and degradation of two transcription factors (10, 11), which has made molecular glue drug be attractive in academic research. Presently, the predominant focus of investigational drugs lies in modifying the surfaces of E3 ubiquitin ligases, aiding the identification and subsequent degradation of target proteins. Owing to its pronounced cytotoxicity against tumor cells, molecular glue has attracted much attention in the field of anticancer drug development (12, 13). Remarkably, molecular glue possesses a modest molecular weight, showcases favorable drug-like properties, and is currently under scrutiny in numerous clinical studies. The ensuing sections will delve comprehensively into the mechanistic intricacies of molecular glue, ongoing strides in drug development, and innovative strategies for designing molecular glue drugs (**Table 1**) (14–16).

**Table 1:** Molecular glue mechanism.

	Molecular Glue name	Ubiquitin Ligase subunit	Target Proteins	Disease
IKZF1/IKZF3 Degrader	Revlimid	CRBN	IKZF1、 IKZF3	Multiple myeloma del(5q), MDS
	Thalidomide	CRBN	IKZF1、 IKZF3	Multiple myeloma
	CC-122	CRBN	IKZF1、IKZF3、ZFP91	NHL、Multiple myeloma、
	CC-220	CRBN	IKZF1、IKZF3、ZFP91、ZNF98	Relapsed/refractory multiple myeloma, Lupus
	CC-99282	CRBN	IKZF1、IKZF3	relapsed/refractory NHL
	CFT7455	CRBN	IKZF1、IKZF3	Multiple myeloma, NHL
GSPT1 Degrader	CC-885	CRBN	GSPT1、CK1α、IKZF1、IKZF3	Acute myeloid leukemia
	CC-90009	CRBN	GSPT1、IKZF1	Relapsed/refractory Multiple myeloma
	BTX-1188	CRBN	GSPT1、IKZF1/3	Acute myeloid leukemia, Myelodysplastic syndrome
	MG-277	CRBN	GSPT1	Cancer
	ZXH-1-161	CRBN	GSPT1、GPST2、CK1α	Multiple myeloma, Acute myeloid leukemia
SALL4 Degrader	Thalidomide	CRBN	SALL4	Multiple myeloma
CDK Degrader	CR8	DDB1	Cyclin K	Cancer
	Glue01	DDB1	Cyclin k	Cancer
CK1α Degrader	FPFT-2216	CRBN	CK1α、IKZF1、IKZF3、PDE6D	Multiple myeloma, Inflammatory diseases
	TMX-4116	CRBN	CK1α	Multiple myeloma
RBM39 Degrader	Indisulam	DCAF15	RNF39、RBM39、RBM23	leukemia
	E7820	DCAF15	RBM39、RBM23	Myelodysplastic syndrome, Acute myeloid leukemia, Solid tumor
BCL6 Degrader	Bl-3802	SIAH1	BCL6	Diffuse large B cell lymphoma
	CCT369260	Na	BCL6	Diffuse large B cell lymphoma
β-catenin Degrader	NRX-1933	SCFβ TrCP	β-catenin	Cancer
	NRX-1532	SCFβ TrCP	β-catenin	Cancer
	NRX-252114	SCFβ TrCP	β-catenin	Colorectal cancer
	NRX-252262	SCFβ TrCP	β-catenin	Colorectal cancer

## 2 E3 ubiquitin ligase-related drug mechanisms

Molecular glues serve as mediators in coordinating protein-protein interactions, particularly when one of the proteins involved is an E3 ligase. In the presence of an E3 ligase, molecular glue instigates a unique interaction between the E3 ubiquitin ligase substrate receptor and the target protein, ultimately resulting in ubiquitination and subsequent degradation of the target protein (17). When the target protein is a tumor pathogenic protein, this process, in turn, imparts a pronounced anticancer effect. Presently, drugs employed in clinical settings or undergoing clinical research predominantly target E3 ubiquitin ligases, including but not limited to CRL4 (Cullin-4-RING E3 ubiquitin ligase), SIAH1, and SCF(β-TRCP).

### 2.1 E3 ubiquitin ligase CRL4

Cullin-RING E3 ligases (CRLs), as important elements of the human ubiquitin-proteasome system, are the largest family of E3 ubiquitin ligases in mammalian cells, regulating substrate specific recognition, ubiquitination, and is a key regulator of the body’s normal function (18–20). The E3 ubiquitin ligase CRL4 is a multifaceted assembly, consisting of the zinc finger domain protein ROC1 (also denoted as RBX1), the Cul4 scaffold protein, and DDB1-associated proteins (21–23), which are mainly involved in biological processes such as cellular DNA damage repair, DNA replication, chromatin remodeling, and maintenance of genome stability (19, 24, 25). It has been found that many molecular glue degraders achieve antitumor efficacy mainly by binding to the substrate receptor proteins, represented by CRBN and DCAF15 (CUL4-associated factor 15),of the CRL4 E3 ubiquitin ligase complex, triggering unprecedented interactions that ubiquitinate and degrade target proteins essential for tumor cell survival (17).

#### 2.1.1 IKZF1/IKZF3 degrader

IKZF1 (Ikaros) and IKZF3 (Aiolos) function as interferon (IFN)-stimulated transcriptional repressors (26, 27). The prompt degradation of IKZF1 and IKZF3 hinders the expression of IRF4 (interferon regulatory factor 4) and c-MYC, leading to the concurrent upregulation of IL-2 (interleukin-2) and IFN-γ (interferon-gamma) in NK (natural killer) cells (28). This orchestrated mechanism mediates potent transcriptional inhibitory effects, resulting in the suppression of tumor cell growth and the induction of apoptosis (29). Notably, studies affirm that the loss or malfunction of IKZF1 is closely linked to drug resistance, heightened relapse rates, and unfavorable prognosis in acute lymphocytic leukemia (ALL) and acute myeloid leukemia (AML) (30–32). A recurring mutation in IKZF3 is identified as a potential cancer driver in chronic lymphocytic leukemia (CLL) (32, 33). Additionally, overexpression of IKZF1/3 is observed in multiple myeloma (MM), which stimulate malignant proliferation of plasma cells (34).

Thalidomide and its derivatives modulate the activity of immune cells, such as T cells and NK cells, by inducing the production of cytokines (IL-2, interferon-gamma, etc.) (35–37). Consequently, they are categorized as immunomodulatory drugs (IMiDs) (38–41). Currently, molecular glue compounds extensively employed in clinical applications predominantly belong to the IMiDs category.

Lenalidomide (Len), another immunomodulator with stronger inhibition of IKZF1 and IKZF3 gene expression, is widely used in the treatment of multiple myeloma (42–44).

**Figure 1:**
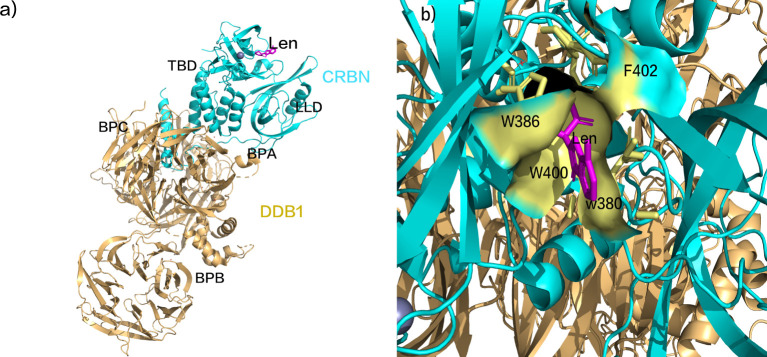
**(A)** Schematic representation of lenalidomide binding to CRBN. **(B)** Schematic representation of the succinimide ring insertion into the TBD hydrophobic pocket. (pdb ID: 4CI2).

The substrate receptor protein of the CRL4, CRBN, comprises an N-terminal Lon-like domain (LLD) reminiscent of the bacterial Lon protease and a C-terminal domain binding thalidomide (TBD). The adaptor protein for the CUL4 ligase, DDB1, provides the binding site for CRBN (**Figure 1A**). The DDB1-binding motif of CRBN encompasses a series of alpha-helices situated on residues 188-248 of LLD. DDB1 consists of three beta-helical domains (BPA, BPB, and BPC), with CRBN binding within a cavity between BPA and BPC (45, 46). The succinimide ring of Len inserts into a hydrophobic pocket composed of three tryptophan residues (W386, W380, W400) and one phenylalanine residue (F402) within TBD (**Figure 1B**) (45, 46). Upon binding, the exposed segment interacts with complementary substrates, thereby modulating ubiquitination and activating diverse downstream anti-tumor responses. In contrast to traditional drugs such as thalidomide and pomalidomide, the next generation of IMiDs shares common adjacent benzene diamine and glutarimide moieties but displays specific distinctions in the glutarimide ring (46–48).

CELMoDs constitute an innovative category of thalidomide analogs, leveraging akin mechanisms for IKZF1/3 degradation. Structurally, both IMiDs and CELMoDs share the succinimide and isoindolinone ring motifs (**Figure 1**). Nevertheless, CELMoDs incorporate supplementary features, including a phenyl ring and a cyclohexyl group, fostering more intricate interactions with CRBN. This heightened interaction induces a significant alteration in CRBN’s protein conformation, augmenting substrate binding affinity and specificity.

**Figure 2:**
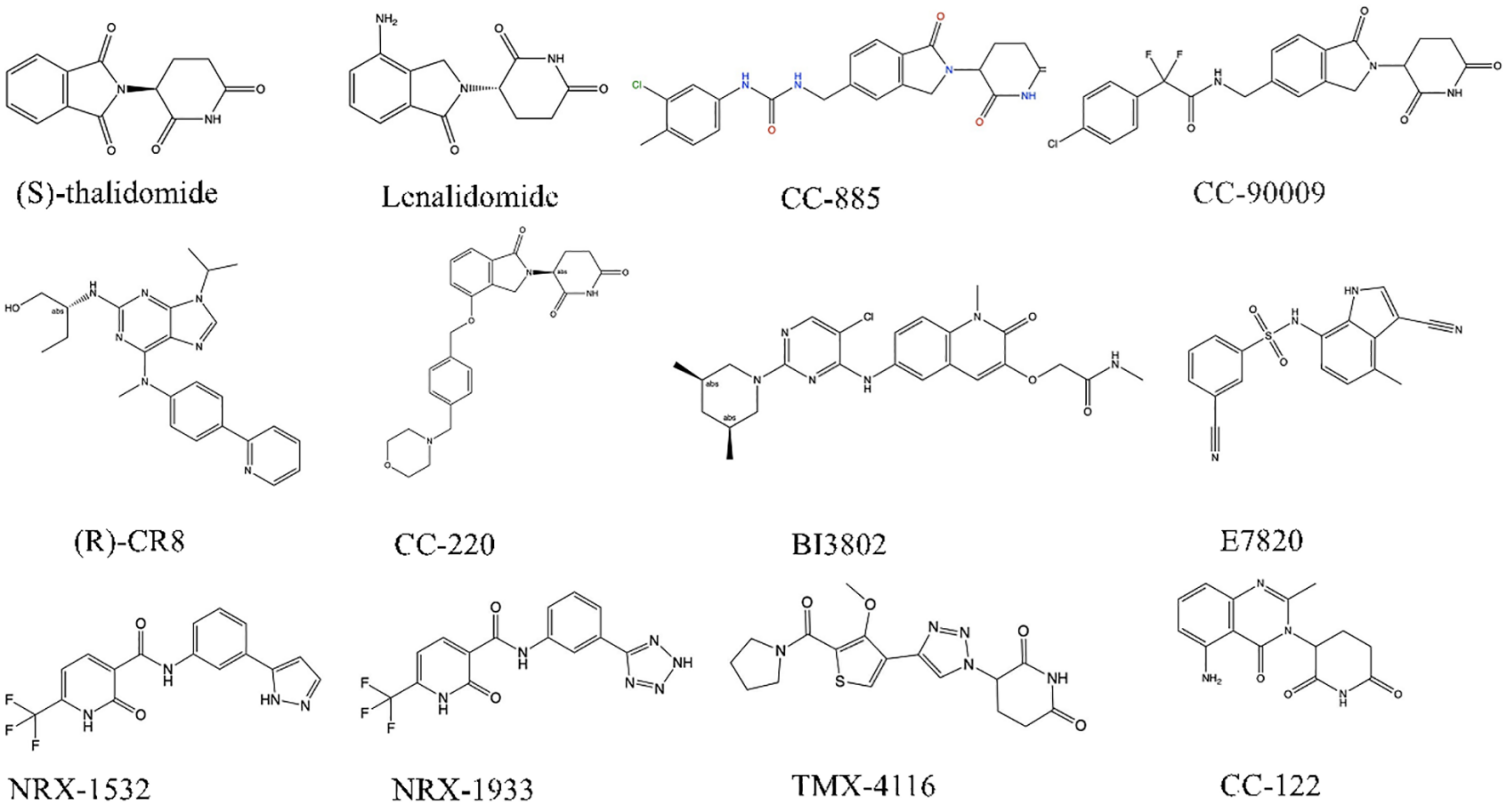
Chemdraw for molecular glue degraders.

Presently, IMiDs find application in treating multiple myeloma, del(5q) myelodysplastic syndrome, non-Hodgkin lymphoma, and various hematologic malignancies (49–51). While in multiple studies utilizing transplanted tumor models, including DL-40(large cell lymphoma cells) (52), CELMoDs have demonstrated a markedly superior tumor inhibition effect in animals compared to IMiDs (53). Based on compounds such as CC-92480, CC-220, CC-99282, and CFT-7455, CELMoDs have emerged as a promising technology in cancer treatment (**Figure 2**).

#### 2.1.2 GSPT1 degrader

**Figure 3:**
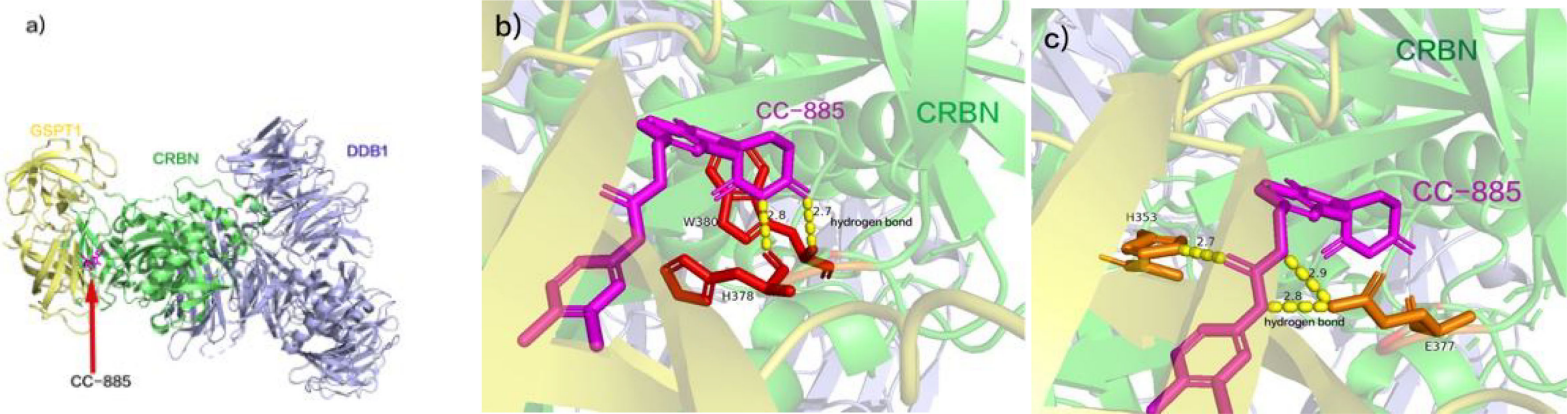
**(A)** CC-885 facilitates the interaction of CRBN with GSPT1. **(B)** CC-885 binds within CRBN’s three Trp pockets, forming three hydrogen bonds with the succinimide ring engaging residues W380 and H378. **(C)** The urea portion of CC-885 forms hydrogen bonds with CRBN residues E377 and H353, and the methyl chlorobenzene ring portion is located near the β-segment of GSPT1 structural domain 3. (pdb ID: 5HXB).

GSPT1 (G1 to S phase transition 1) functions as a crucial release factor in translation termination (54), contributing to diverse cellular processes such as cell cycle progression (55), cytoskeletal organization (56), and apoptosis (57, 58). Its overexpression is noted across various tumors, including gastric, breast, liver cancers (59–61), and AML. The molecular glue drug CC-885 facilitates the interaction of CRBN with the novel substrate GSPT1, resulting in its degradation and imparting anti-proliferative effects in AML. Researchers have delineated the CC-885-mediated CRBN-GSPT1 interaction at the crystal structure level (**Figure 3A**) (62). Specifically, GSPT1 domain 3 docks at the CC-885 binding site, directly interacting with both CC-885 and CRBN surfaces. CC-885 binds within CRBN’s three Trp pockets, forming hydrogen bonds (the hydrogen bond lengths are 2.7 Å and 2.8 Å, respectively) with the succinimide ring engaging residues W380 and H378 (**Figure 3B**) (62). The isoindolinone ring of CC-885 interacts with both CRBN and GSPT1. CC-885’s expanded chemical structure enables further interaction with CRBN and GSPT1. Its urea portion forms hydrogen bonds with CRBN residues E377 and H353 (the three hydrogen bond lengths are 2.7 Å, 2.8 Å, and 2.9 Å, respectively), while its methyl chlorobenzene ring, positioned near the β-segment of GSPT1 domain 3, facilitates additional interactions, mediating GSPT1 degradation (**Figure 3C**) (62). Despite its capability to degrade CRBN substrates IKZF1, IKZF3, and CK1α, leading to off-target effects, the clinical development of CC-885 was terminated. Nevertheless, the identification of the novel substrate GSPT1 expands the clinical application potential of CELMoDs. The elucidation of the crystal structure of the CRBN-DDB1-GSPT1-CC-885 complex establishes a foundation for uncovering new CRBN substrates. Notably, CC-90009, designed as a selective GSPT1 degrader with a 2-fluoromethyl substitution for the nitrogen atom in CC-885’s benzamide, represents the first clinical-grade CELMoD (63). In comparison to CC-885, CC-90009 exhibits dose-dependent efficacy in degrading GSPT1 and IKZF1, demonstrating increased selectivity for GSPT1 and improved safety (64, 65). Currently, A phase Ib clinical trial of CC-90009 in combination with venetoclax and azacitidine for acute myeloid leukemia is ongoing (64, 65).

#### 2.1.3 SALL4 degrader

Sal-like protein 4 (SALL4) acts as a transcription factor, contains the Cys2His2 zinc finger (C2H2-ZF) domain predominantly present in embryonic stem cells, playing a pivotal role in their self-renewal and differentiation. Throughout individual development, its expression gradually diminishes in most adult tissues, eventually reaching a silent state. Nevertheless, its reactivation in tumor cells significantly contributes to the initiation and progression of tumors (66–68). SALL4 exhibits notable overexpression in diverse cancers, such as colorectal, lung, breast cancer, and acute myeloid leukemia, establishing close associations with tumor cell proliferation, apoptosis, invasion, and drug resistance (69).

Thalidomide, a well-known drug, comprises two enantiomers, S(-) and R(+) (70, 71). (R)-thalidomide is recognized for alleviating morning sickness, vomiting, and inducing sedation (72), whereas (S)-thalidomide exhibits strong teratogenic effects (73). Intriguingly, the teratogenicity of (S)-thalidomide serves as a molecular glue degrader, targeting the degradation of SALL4 during the embryonic period (74).

**Figure 4:**
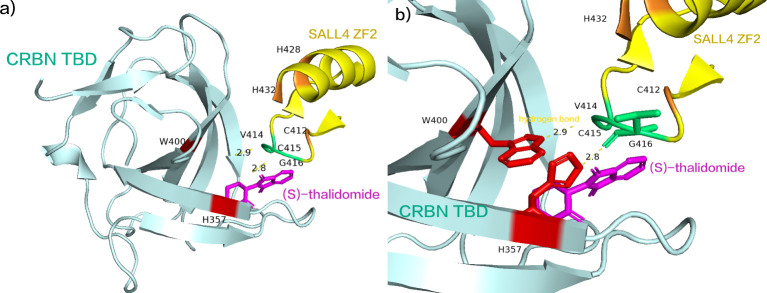
**(A)** (S)-thalidomide simultaneously binds SALL4 ZF2 and CRBN TBD. **(B)** the side chains of W400 and H357 in CRBN TBD form direct hydrogen bonds with the main chain carbonyls on the β-hairpin ring of SALL4 ZF2 (V414, C415, and G416). (pdb ID: 7BQU).

A recent study has elucidated that enantiomeric (S)-thalidomide promotes the interaction between SALL4 and CRBN (**Figure 4A**). The thalidomide-binding domain (TBD) of SALL4 ZF2 emerges as an evolutionarily conserved positive selection site. SALL4 ZF2, featuring the typical C2H2 ZF domain, comprises β-hairpin structures (β1’ and β2’) and α-helical structures (α1’), connected by the Zn2+-binding conserved CXXC and HXXXH motifs (C412, C415, H428, and H432) (75). SALL4 ZF2 establishes contact with the open face of the twisted β-sheet of CRBN TBD, and in the 5HT-mediated complex structure, the β-hairpin ring aligns seamlessly with respect to the position of CRBN TBD. Notably, the side chains of W400 and H357 in CRBN TBD form direct hydrogen bonds (**Figure 4B**, the two hydrogen bond lengths are 2.8 Å, 2.9 Å, respectively) with the main chain carbonyls on the β-hairpin ring of SALL4 ZF2 (V414, C415, and G416) (75). Subsequently, binding to CRBN in the E3 ubiquitin ligase promotes ubiquitination of SALL4 on the C2H2-ZF domain, leading to its subsequent degradation (75). Presently, numerous molecular glue drugs in clinical trials are strategically designed based on the thalidomide structure (48, 76). The comprehensive elucidation of the mechanism and structural alterations of thalidomide not only enhances our understanding but also provides valuable insights for future drug development grounded in the thalidomide structure.

#### 2.1.4 CDK degrader

Cell cycle cyclin-dependent kinases (CDKs) intricately regulate both the cell cycle and gene transcription. CDK12 assumes a critical role in transcriptional regulation by promoting the phosphorylation of RNA polymerase II, thereby influencing RNA splicing, DNA damage response, and maintaining genomic stability (77–79). It has been found that CDK12 is overexpressed in various tumors, such as breast cancer, ovarian cancer, and prostate cancer, which could be a potential anti-tumor target (80–85).

The CDK12 molecular glue inhibitor, (R)-CR8, was identified through a comprehensive analysis correlating the cytotoxicity of over 4000 small molecules in diverse cancer cell lines with the expression levels of E3 ligases in a database (86, 87). Notably, CRL4 E3 ligase-related molecular glue exhibits two common modes: one reliant on CRBN and the other on DCAF15 (88–90). These modes share the DDB1-CUL4-RING-E2 complex, diverging at the receptor protein. Remarkably, (R)-CR8 exhibits the unique ability to recruit Cyclin K to form a ternary complex, cyclin K- CDK12-CR8, without necessitating a standard substrate receptor (bypassing CRBN or DCAF15) (91). This induces the ubiquitination of CDK, resulting in its degradation through the proteasome system and subsequent apoptosis in tumor cells.

**Figure 5:**
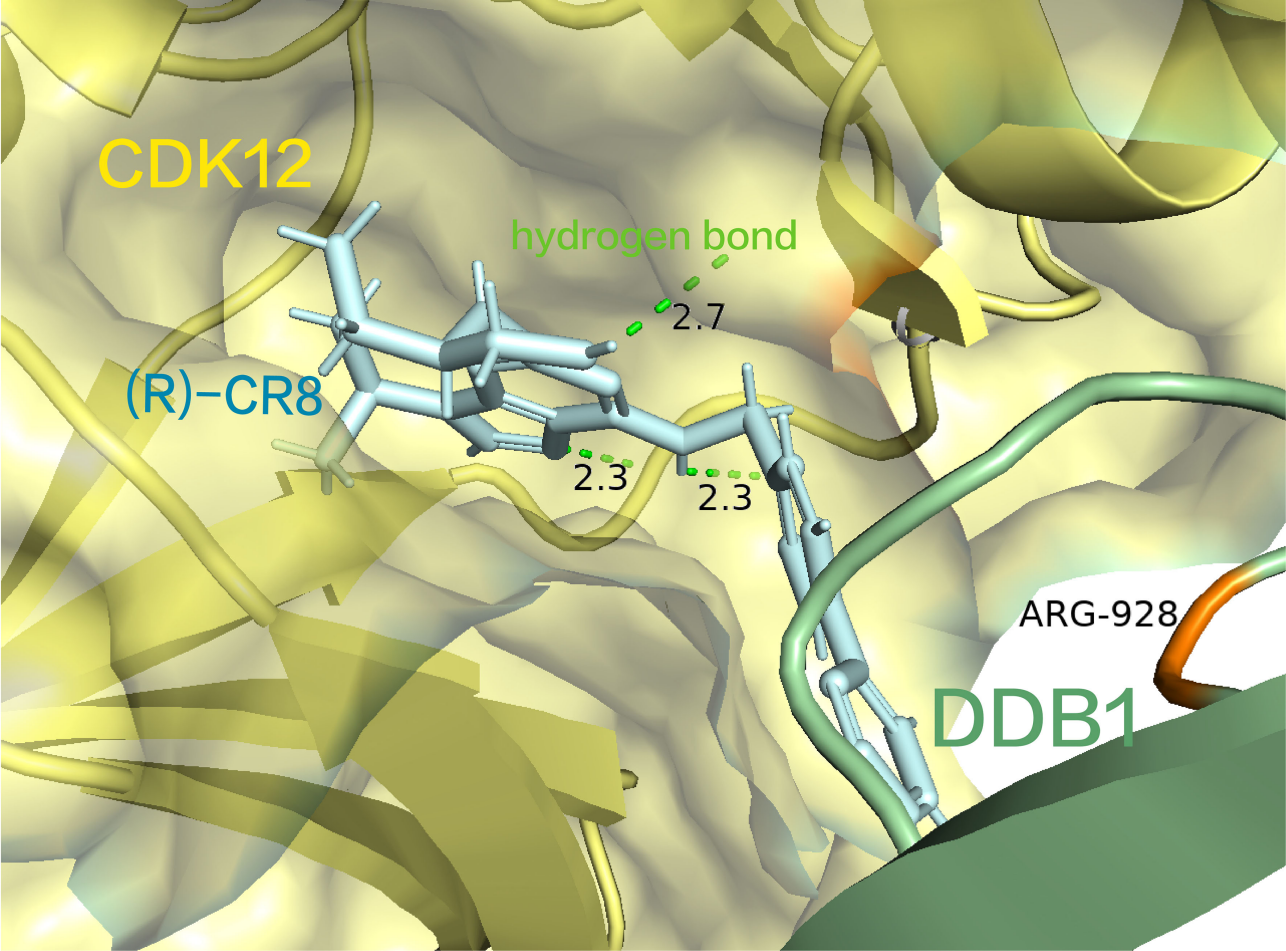
(R) -CR8 occupies the ATP-binding pocket of CDK12, forming critical hydrogen bonds with the purine structure and a cationic interaction with R928 in the BPC structural domain of DDB1. (pdb ID: 6TD3).

Structurally, (R)-CR8 strategically occupies the ATP-binding pocket of CDK12, forming essential hydrogen bonds with the purine structure in the hinge region of CDK12(the three hydrogen bond lengths are 2.3 Å, 2.3 Å, and 2.7 Å, respectively) (91). Additionally, it establishes two π-cation interactions with the R928 in the BPC domain of DDB1 (**Figure 5**) (91). This interaction is facilitated by its solvent-domain oriented hydrophobic phenylpyridine ring, resulting in the formation of a CDK12-Cyclin K and CUL4 bridging protein DDB1 (RING BOX protein 1) complex (91). At present, (R)-CR8 is in the preclinical stage.

#### 2.1.5 CK1α degrader

Casein kinase 1 alpha (CK1α), belonging to the CK1 protein family, plays a crucial role in regulating signaling pathways associated with autoimmune diseases, neurodegenerative disorders, and cancers (92–94). It was found that targeting CK1α disrupts CK1α/β-catenin signaling, could effectively controlling the growth and survival of organoid-like structures linked to developmental abnormalities in mice and humans (95, 96). These abnormalities indicate an increased risk of cancer (97). Moreover, infrequent CK1α mutations have been identified in various solid tumors and hematological malignancies, suggesting that CK1α may be an anti-cancer therapeutic target (94, 98).

The triazole compound FPFT-2216 induces the formation of a ternary compound involving the E3 ubiquitin ligase CUL4-DDB1-CRBN and CK1α, leading to CK1α ubiquitination and degradation (99). However, FPFT-2216 displays non-specific degradation activity, impacting not only known targets like IKZF1, IKZF3 and CK1α, but also PDE6D (100). This lack of drug selectivity has caused potential safety concerns. In contrast, TMX-4116, a molecular glue degrader derived from FPFT-2216, lacks a cyclic amide group (99). This structural distinction imparts selective degradation activity against CK1α. Cellular assays have validated that TMX-4116 significantly degrades CK1α in acute lymphoblastic leukemia cells (MOLT4), human T lymphoblast cells (Jurkat), and MM cells (MM.1S), without affecting PDE6D, IKZF1, and IKZF3 (99). This enhanced degradation selectivity positions TMX-4116 in the preclinical research stage for multiple myeloma treatment.

#### 2.1.6 RBM39 degrader

RNA-binding motif protein 39 (RBM39) is an RNA-binding protein involved in RNA splicing and transcriptional regulation (101, 102). Elevated RBM39 levels contribute to aberrant splicing events and differential gene expression (103). In liver cancer cells, increased arginine uptake is associated with RBM39, sustaining a higher oncogenic metabolism (101, 104). Additionally, in neuroblastoma, RBM39 acts as a crucial splicing factor stimulating malignancy (105). Degrading RBM39 disrupts abnormal splicing through splice dysregulation types, such as intron retention and exon skipping, resulting in anti-tumor activity (106).

**Figure 6:**
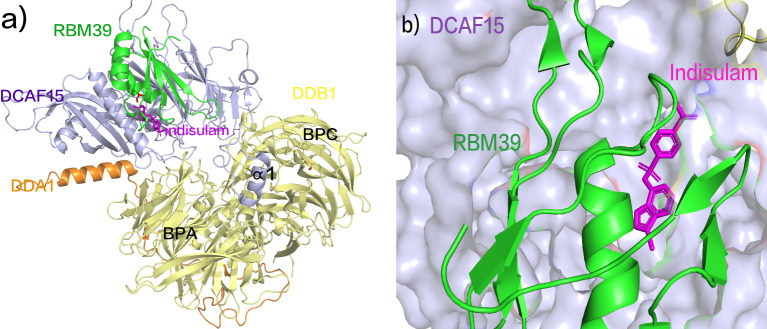
**(A)** Crystal Structure of DCAF15 DDB1 DDA1 indisulam RBM39 Composite. **(B)** Indisulam binds to a shallow pocket on the surface of DCAF15, forming a modified interface that interacts with RBM39 through the α1 helix of the RRM2 domain (pdb ID: 6SJ7).

Indisulam (E7070) relies on recruiting the splicing protein RBM39 to CUL4-DCAF15, acting as a molecular glue between E3 ubiquitin ligase CUL4-DDB1-DCAF15 and RBM39 to facilitate RBM39 degradation (106). Despite its relatively mild affinity for DCAF15, the cooperative binding of both Indisulam and RBM39 to DCAF15 allows the construction of a stable ternary complex (**Figure 6A**) (107). Indisulam binds to a shallow pocket on the surface of DCAF15, forming a modified interface that interacts with RBM39 through the α1 helix of the RRM2 domain (**Figure 6B**), ultimately leading to RBM39 ubiquitination and degradation (108). Studies have demonstrated significant regression of tumor tissue in a high-risk neuroblastoma xenograft mouse model with MYCN overexpression and ALK F1178L mutation upon Indisulam treatment (107). However, Indisulam exhibits inferior oral bioavailability and strong myelosuppression effects. In contrast, E7820, a novel aryl sulfonamide molecular glue, recruits DCAF15 to degrade RBM39 (90, 109). Unlike Indisulam, E7820 demonstrates favorable oral bioavailability and is currently in Phase II clinical trials (NCT05024994) for relapsed or refractory AML, MDS, or CMML (110).

### 2.2 E3 ubiquitin ligase SIAH1(BCL6 degrader)

The E3 ubiquitin ligase Siah1, a RING-type E3 ubiquitin ligase, plays a pivotal role in protein ubiquitination, contributing to the degradation of key targets such as Tribbles 3 homolog (TRB3) and BCL6 (111). BCL6, functioning as a widely expressed transcriptional repressor, inhibits genes involved in cell cycle control, apoptosis, and differentiation, with selective growth suppression observed in BCL6-driven lymphoma cell lines (112, 113). Consequently, BCL6 emerges as a promising therapeutic target for anticancer treatment.

**Figure 7:**
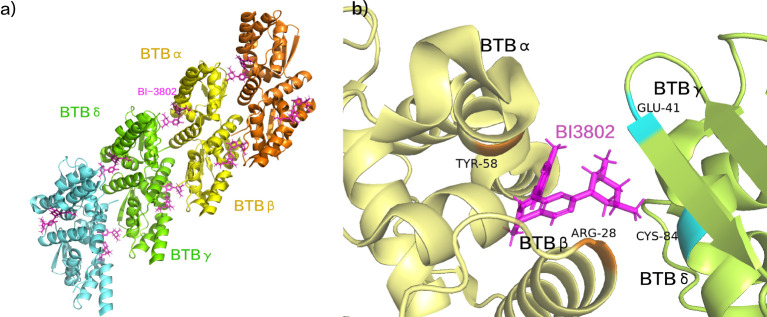
**(A)** BI-3802 induced BCL6 aggregation. **(B)** The interaction between BI-3802 and BTB structural domains. (pdb ID: 6XMX).

The molecular glue degrader BI-3802 leverages the E3 ubiquitin ligase SIAH1 to induce the degradation of BCL6 (114). This process focusses on the specific targeting of the Broad complex, Tramtrack, and Bric-à-brac (BTB) domain of BCL6 (115, 116). The 3,5-dimethylpiperidine component interacts with another molecule of the BTB domain homodimer, instigating BCL6 aggregation and the formation of higher-order BCL6 sinusoidal protein filaments (**Figure 7A**) (117). Specifically, BI-3802 establishes direct contact with Tyr58 of BTBα within the groove between BCL6 homodimers, engaging in a hydrophobic interaction with Cys84 of the adjacent BCL6 homodimer (BCL6γ/δ) (117). Furthermore, BI-3802’s binding to BCL6 homodimers facilitates the creation of a salt bridge between Arg28 of BTBβ and Glu41 of BTBγ, resulting in enhanced affinity for SIAH1 (**Figure 7B**) (117). The E3 ubiquitin ligase SIAH1 coordinates the degradation of aggregated proteins induced by BI-3802 through acting on the VxP motif of the BCL6 protein, located specifically at residues 249-251 of BCL6 (117). BI-3802 promotes the interaction between the BCL6 protein and SIAH1, thereby expediting the ubiquitination and degradation of BCL6 protein. This process culminates in a noteworthy inhibition of BCL6 target genes and imparts anti-proliferative effects in DLBCL cell lines (117). In comparison to traditional BCL6 inhibitors, BI-3802, as a small molecule, induces aggregation alongside highly specific protein degradation, showcasing superior pharmacological activity.

### 2.3 E3 ubiquitin ligase SCF(β-TRCP)(β-catenin degrader)

The aberrant activation of the Wnt signaling pathway is implicated in the pathogenesis of diverse tumors, including non-small cell lung cancer, pancreatic cancer, and breast cancer (52). Consequently, modulating Wnt signaling, either by downregulating or restoring its excessive activation, holds therapeutic promise for cancer treatment. In normal cells, β-catenin undergoes recognition by the E3 ubiquitin ligase SCFβ-Trcp through phosphorylated Ser33 and Ser37, resulting in β-catenin degradation and maintaining it at lower levels. Conversely, colorectal cancer cells exhibit attenuated effective binding of β-catenin to β-TrCP, leading to its stabilization and augmentation of oncogenic transcription programs (118).

**Figure 8:**
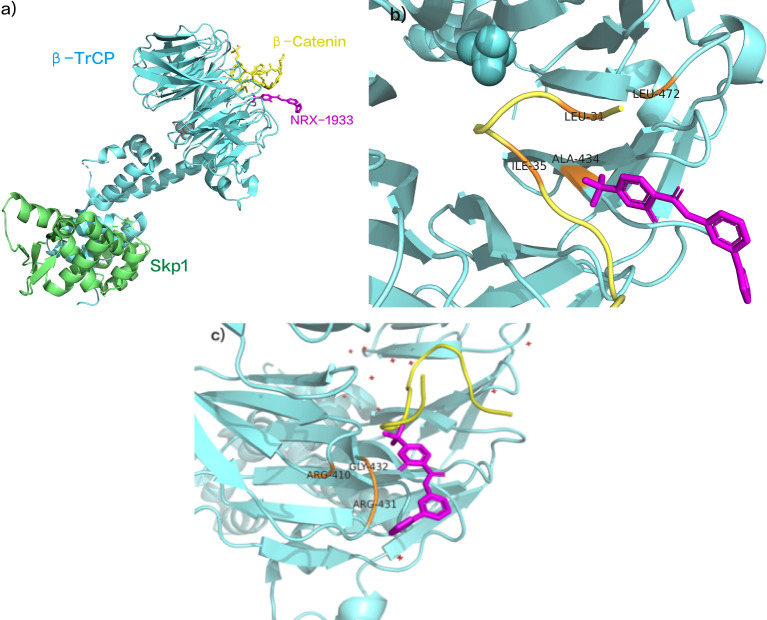
**(A)** NRX-1933 as SCF β- Trcp and β- Catenin intermolecular glue. **(B)** NRX-1933 combined with β- Catenin: β- TrCP interface. **(C)** NRX-1933 Enhancement β- Catenin: β- TrCP interaction. (pdb ID:6M93).

NRX-1933, identified as a molecular glue degrader, enhances the interaction between β-catenin and β-TrCP (**Figure 8A**). A study elucidated the structural mechanism by which NRX-1933, as a molecular glue-type drug, degrades β-catenin (118). Initially, NRX-1933 binds to the β-catenin:β-TrCP interface, where trifluoromethylpyridinone occupies a small pocket exposed due to the absence of phosphorylation at Ser37 (119). The trifluoromethyl substituent fills a hydrophobic pocket formed by Leu31 and Ile35 residues of the β-catenin peptide and Ala434 and Leu472 residues of β-TrCP, establishing crucial hydrophobic interactions with both β-catenin and β-TrCP (**Figure 8B**). Specifically, trifluoromethylpyridinone occupies the phosphorylation site of Ser37 on β-catenin, mimicking phosphorylation by forming a hydrogen bond with the main-chain N-H of Gly432 on β-TrCP. Finally, the phenyltetrazole tail group extends outward from the β-catenin: β-TrCP interface, with the phenyl ring overlapping with Arg431 of β-TrCP, and the anionic tetrazole adjacent to the cationic residues Arg410 and Arg431 of β-TrCP (**Figure 8C**) (118). This significantly enhances the binding affinity of the β-catenin: β-TrCP interface, ultimately leading to ubiquitination and degradation of β-catenin. Although NRX-1933 has not been developed into clinical trials, its reasonable molecular weight and physicochemical properties make it a promising starting point for structural design in the development of novel anti-cancer drugs.

Simonetta et al. identified the molecular glue NRX-1532 through high-throughput screening, to restore the binding between non-phosphorylated β-catenin and CRL1β-TrCP. After structure optimization based on the binding interface, the drug activity of NRX-1532 is significantly improved. NRX-252114, a novel drug developed based on the compound NRX-1532, is intriguing as it restores the interaction between mutated β-catenin (typically unable to bind to β-TrCP) and β-TrCP, a natural E3 ubiquitin ligase. This interaction induces proteasomal degradation of mutated β-catenin, exerting anti-tumor effects. Both NRX-1532 and NRX-252114 are currently in the preclinical research stage.

## 3 Progress of clinical trials

Currently, numerous clinical trials are in progress to evaluate the tolerance, safety, pharmacokinetics, and various characteristics of molecular glue drugs in patients with solid tumors, hematologic malignancies, and immune system disorders (**Table 2**).

**Table 2:** Clinical trials stage drugs.

Molecular glue name	Target proteins	Clinical trial stage	Clinical trial number	R&D institutions	Indications
ICP-490	IKZF1/3	Phase I/II	NCT05719701	InnoCare Pharma Limited	Relapsed/refractory multiple myeloma
SP-3164	IKZF1/3	Phase I	NCT05979857	Salarius Pharmaceuticals, Inc.	Relapsed/refractory NHL
CC-92480	IKZF1/3	Phase I/II/III	NCT05389722 NCT04211545 NCT04839809 NCT03803644 ……	Bristol Myers Squibb	Recurrent/refractory multiple myeloma
CC-99282	IKZF1/3	Phase I/II	NCT03930953 NCT06108232 NCT04884035 NCT04434196 ……	Bristol Myers Squibb	NHL
CC-122	IKZF1/3	Phase I/II	NCT02509039; NCT02406742; NCT02859324; NCT01421524 ……	Bristol Myers Squibb	Chronic lymphocytic leukemia、Advanced solid tumor、 NHL/Multiple myeloma
CFT-7455	IKZF1/3	Phase I/II	NCT04756726	Therapeutics	Relapsed/refractory NHL 、Relapsed/refractory multiple myeloma、
KPG-818	IKZF1/3	Phase I/II	NCT04643067 NCT04283097 NCT03949426	Kangpu Biomedical Technology (Shanghai)Co., Ltd.	Systemic lupus Erythematosus/hematological malignancy
CC-90009	IKZF1/3, GSPT1	Phase I/II	NCT02848001 NCT04297124 NCT04336982	Bristol Myers Squibb	Relapsed/refractory acute myeloid leukemia、Recurrent refractory high-risk myelodysplastic syndrome
NX-2127	IKZF1/3 BTK	Phase I	NCT04830137	Nurix Therapeutics	Relapsed/refractory B-cell malignant tumors
CC-220	IKZF1/2/3	Phase I/II/III	NCT02773030 NCT03135509 NCT01733875 NCT02034773 ……	CelgeneBristo1-Myers Squibb)	Systemic lupus erythematosus、NHL、Multiple myeloma
DKY-709	IKZF2	Phase I	NCT03891953	Novartis	Advanced solid tumors
BTX-1188	IKZF1/3 GSPT1	Phase I	NCT05144334	BioTheryX	Acute myeloid leukemia, Myelodysplastic syndrome
MRT-2359	GSPT1	Phase I/II	NCT05546268	Monte Rosa Therapeutics, Inc.	Diffuse B-cell lymphoma of lung cancer
BAY2666605	PDE3A SLFN12	Phase I	NCT04809805	Bayer	Advanced malignant tumors of skin cancer
E7070	RBM39 RBM23 RNF39	Phase I/II	NCT00003976; NCT00003981;NCT00165594;NCT00014625 ……	Eisai Co Ltd.	Relapsed AML、High-risk myelodysplastic syndrome、Solid tumor
E7820	RBM39 RBM23	Phase I/II/III/IV	NCT05024994 NCT00078637 NCT01347645 NCT01133990 ……	Eisai Co Ltd.	Myelodysplastic syndrome, Acute myeloid leukemia, Solid tumor

### 3.1 CC-220 (iberdomide)

CC-220 (Iberdomide), a novel drug developed by Celgene Corporation, represents a new generation of oral CELMoDs. It functions by inducing the degradation of the transcription factors IKZF1 and IKZF3 while inhibiting the production of tumor necrosis factor alpha (TNF-α) (120). According to published clinical research data, iberdomide has demonstrated promising therapeutic effects in the treatment of relapsed/refractory multiple myeloma (RRMM) (121).

There are two ongoing clinical trials investigating the novel drug CC-220: the phase 1/2 CC-220-MM-001 (NCT02773030) and the phase 3 EXCALIBER-RRMM (NCT04975997) trials. In the initial phase of CC-220-MM-001, Iberdomide, Bortezomib, and Dexamethasone were administered to transplant ineligible patients with newly diagnosed multiple myeloma (NDMM), demonstrating favorable efficacy with sustained profound responses. The overall response rate (ORR) within the assessed population reached 100%, with 87.5% achieving ≥ very good partial response (VGPR) and 56.25% achieving ≥ complete response (CR). Among those achieving ≥ VGPR, 43% (6/14) attained minimal residual disease (MRD) negativity, and 68.8% experienced responses within a 6-week timeframe (122). These findings provide support for the continued exploration of Iberdomide (IBER) in MM. When administered in combination with Dexamethasone (DEX), the recommended dose for the subsequent stage of IBER was 1.6 mg (123).

In the dose expansion phase of the CC-220-MM-001 trial, the IBER + DEX combination exhibited notable efficacy in heavily treated, triple-class-exposed, and refractory patients with RRMM, including those previously subjected to anti-BCMA therapies. The ORR was 26.2%, the clinical benefit rate (≥ minimal response) was 36.4%, and the disease control rate (≥ stable disease) was 79.4%. A significant proportion of patients (82.2%) reported grade 3-4 treatment-emergent adverse events (TEAE), predominantly hematologic TEAE, encompassing neutropenia (44.9%), anemia (28.0%), thrombocytopenia (21.5%), and leukopenia (20.6%) (123). The incidence of grade 3-4 non-hematologic TEAE was comparatively lower. In general, these patients exhibited a preserved health-related quality of life (HRQoL). The combination therapy of IBER and DEX for RRMM is slated for comparison with DRD in the phase 3 EXCALIBER-RRMM (NCT04975997) trial (123).

Based on preclinical research data, Iberdomide is presently undergoing phase 3 clinical trials for two indications: second-line (2L) treatment for MM and maintenance therapy for NDMM patients following autologous stem cell transplantation.

### 3.2 CC-122 (Avadomide)

CC-122 (Avadomide)also belongs to the CELMoD class of drugs, which can induce the degradation of IKZF1 and IKZF3 through the proteasome pathway (124), exhibiting anti-lymphoma, anti-angiogenesis, and immunomodulatory effects, making it a promising new-generation therapeutic agent for the treatment of non-Hodgkin’s lymphoma (NHL) (125–127).

The Phase I clinical trial of Avadomide (NCT01421524) focused on various solid tumors, NHL, and MM. In NHL patients, the objective response rate (ORR) reached 60%, with 50% of RRMM patients showing stable disease. The primary adverse reactions, fatigue, and neutropenia demonstrated acceptable safety and tolerability. These results provided the basis for the subsequent Phase Ib study, CC-122-NHL-001 (NCT02417285). This Phase Ib study assessed the safety and tolerability of Avadomide in combination with Obinutuzumab for treating patients with relapsed/refractory follicular lymphoma (R/R FL), along with the recommended Phase II dosage. In the dose expansion phase, the ORR for patients reached 71%, with 40% achieving a complete response (CR). The Recommended Phase 2 Dose (RP2D) was determined as 3 mg capsules in combination with 1000 mg Obinutuzumab. Overall, Avadomide exhibited manageable safety and durable responses (128, 129).

CELMoD class drugs, including CC-220 (iberdomide) and CC-122 (Avadomide), have been verified for acceptable safety and pharmacokinetics in patients with solid tumors, NHL, and MM.

The fourth-generation drug, CC-99282, not only induces profound (>90%) and sustained degradation of Ikaros and Aiolos in immune cells but also shows a lower incidence of severe febrile neutropenia, ensuring good safety. The new-generation degrader, GLB-002, achieves highly selective degradation of IKZF1/3 proteins, demonstrating promising anti-proliferative activity *in vitro* in resistant cell lines of NHL and MM. This solves the challenges associated with non-specific degraders targeting IKZF1/3, such as poor selectivity, off-target toxicity, and resistance. GLB-002, a highly promising drug for hematologic malignancies as NHL and MM, was approved for clinical trials by the National Medical Products Administration (NMPA) in July 2023(Acceptance number: CXHL2300799).

### 3.3 CC-99282(golcadomide)

The CELMoD drug CC-99282 (Golcadomide) triggers the proteasome pathway to degrade IKZF1 and IKZF3, thereby exhibiting antitumor effects (130). *In vitro* studies have demonstrated that CC-92480 potentiates anti-proliferative and tumoricidal activity in MM cell lines, even in those resistant to lenalidomide and pomalidomide, while also displaying robust immunostimulatory properties (131). Presently, a Phase 1 clinical trial is being conducted to evaluate its therapeutic potential in non-Hodgkin’s lymphoma.

The CC-99282-NHL-001 study (NCT03930953) is a multicenter Phase I clinical trial designed for relapsed/refractory non-Hodgkin’s lymphoma (R/R NHL) patients who have received multiple prior treatments. This trial represents the inaugural human study, including a dosage escalation segment for individual therapy (Part A) and a dosage escalation phase with or without combination therapy with rituximab (Part B) (132). In Part A, individual therapy demonstrated notable efficacy among heavily treated R/R NHL patients, with an overall response rate of 43%, with a 17% complete response rate (132). For FL patients, the overall response rate reached 75%, with 38% achieving complete response. Golcadomide demonstrated reassuring safety, with 64% of patients experiencing neutropenia, within the expected range. In Part B, the golcadomide and rituximab combination demonstrated an overall response rate of 43%, the median duration of response (DOR) is 299 days for DLBCL patients and 448 days for FL patients (132). Presently, BeiGene’s Investigational New Drug application for CC-99282 capsules has received approval from NMPA, indicating its potential for advancing into clinical development.

### 3.4 E7070 (Indisulam)

The RBM39 protein plays a crucial role in maintaining AML by disrupting the alignment of the HOXA9 target gene (133). Furthermore, deleting RBM39 has been observed to modify the splicing of mRNA, which is essential for AML cell proliferation (134). Consequently, targeting RBM39 for degradation emerges as a potential therapeutic strategy for treating AML and other cancers. Notably, E7070(Indisulam) selectively induces the ubiquitination and degradation of the RBM39 protein, thereby exhibiting anti-AML effects. Presently, this compound is undergoing phase II clinical trials (135).

E7070 exhibited no solitary activity against leukemia, thus, in a Phase II trial (NCT01692197), it was combined with idarubicin and cytarabine for treating R/R AML and high-risk myelodysplastic syndrome (MDS) patients. In 40 patients, 17 (43%) had diploid cytogenetics, and 13 (33%) manifested -5/-7 abnormalities. Ultimately, 11 cases (35%) attained complete remission (CR) or CR with incomplete count recovery (CRi) (135). Seven responders subsequently received stem cell transplantation (SCT). The most prevalent non-hematologic toxicities of Grade ≥ 3 were electrolyte abnormalities (50%) and febrile neutropenia (28%) (135). Overall, the combined therapy of E7070 with idarubicin and cytarabine proved advantageous for R/R AML and high-risk MDS patients, exhibiting good tolerability and promising efficacy in heavily treated AML/MDS patients.

### 3.5 E7820

Similar to Indisulam, E7820 also acts against AML by targeting RBM39 protein for ubiquitination degradation (109). The Phase II clinical trial of E7820 (NCT05024994) focuses on adults with relapsed/refractory myeloid malignancies, which have mutations like SF3B1, SRSF2, U2AF1 (136). Employing the optimal Simon 2-stage design, the trial reports ORRs of 10% and 30%, indicating less promising outcomes (136). E7820 demonstrates good tolerability with relatively low hematologic toxicity, and no treatment discontinuation due to drug-related adverse events was observed. Administered as a monotherapy at the maximum tolerated dose established in a previous trial (NCT01773421), E7820 exhibits acceptable safety profiles (110). Despite E7820’s limited efficacy, evidence targeting splicing factor-mutated diseases, particularly through RBM39 degradation, emphasizes its potential in managing human malignancies.

### 3.6 CC-90009

CC-90009 binds to CRL4CRBN, selectively targets GSPT1 ubiquitination and proteasome degradation, and promotes rapid apoptosis of AML cells by effectively eliminating GSPT1 (137). In primary leukemia xenotransplantation models, leukemia implantation and leukemia stem cell (LSC) counts were significantly reduced (64). These findings demonstrate that CC-90009 can be used in clinical or preclinical studies.

CC-90009 is presently under investigation in patients diagnosed with AML for its efficacy as a monotherapy (NCT02848001). CC-90009 treatment induces a rapid decrease in peripheral and bone marrow progenitor cells, indicating preliminary promise in patients with relapsed or refractory AML. Subsequently, the combined efficacy of CC-90009 with venetoclax (VEN)/azacitidine (AZA) is being evaluated in a Phase 1/2 trial involving AML patients (NCT04336982). The aim of this trial is to evaluate the safety, tolerability, pharmacokinetics (PK), and recommended Phase II dose (RP2D) of CC90009 in combination with anti-leukemic drugs for AML treatment, with an anticipated completion by 2025. Another potent, selective, and orally bioavailable GSPT1 degrader, MRT-2359, has entered Phase I/II clinical trials (NCT05546268) for treating myc-driven and other specified solid tumors, such as lung cancer and diffuse large B-cell lymphoma. However, to date, no selective GSPT1 degraders have received clinical approval.

## 4 Design strategies for novel molecular glues

At present, the identification of pharmacologically active novel molecular glues is predominantly serendipitous. However, as researchers consistently to explore reasonable design methods for converting protein-targeting ligands into molecular glue degraders, a series of potential candidate molecules have attracted much attention.

### 4.1 Linking specific chemical handles

Coupling biologically active compounds to specific chemical handles for site-specific coupling to improve drug affinity is a commonly used approach for drug development (138, 139). Therefore, researchers have applied it to the development of new drugs as molecular glue degraders.

Research chose the CDK4/6 inhibitor Ribociclib as the foundational molecule, introducing various derivatives of “chemical handles” into the solvent-accessible region of Ribociclib, resulting in the creation of nine Ribociclib analogs (140, 141). Initially, the investigation pinpointed the small molecule EST1027, derived from trifluoromethylphenyl cinnamoyl amide, for its notable capability to degrade CDK4 (140). Subsequent inquiries disclosed that the degradative potential of both EST1027 and its analogue, EST1060, necessitates the existence of a covalent bond. Employing probes to identify proteins covalently bound to EST1027, the researchers figured out one E3 ligase, RNF126, intricately connected to the ubiquitin proteasome system (140). Consequently, commencing from the structures of EST1027 and EST1060, the authors incrementally expanded the functional groups on EST1027, conclusively establishing that the p-methoxyphenylpyrimidinyl fumarate structure constitutes the minimal unit proficient in covalently binding with RNF126 (140). Upon identifying JP-2-196 as the minimal covalent binding unit for the E3 ubiquitin ligase RNF126, the researchers fused this minimal covalent structure with diverse target protein ligands, yielding molecular glue degraders efficacious against various targets, including CDK4, BCR-ABL, c-ABL, PDE5, BRD4, AR, AR-V7, BTK, SMARCA2, LRRK2, and others (140). In conclusion, the strategic transformation of protein ligands into protein degraders through the linkage of specific chemical handles has proved to be an innovative approach in molecular glue design.

### 4.2 Harnessing natural products

Both PROTACs and molecular glue degraders induce E3 ubiquitin ligases to approach the target protein, leading to ubiquitination of the protein and degradation of the target protein in a proteasome-dependent manner. PROTACs are relatively modular in design, consisting of the target protein-targeting ligand attached to the E3 ubiquitin ligase ligand by a “linker” (142, 143). The design of PROTAC is relatively modular, consisting of a target protein ligand attached to an E3 ubiquitin ligase ligand via a “linker”. The discovery of novel molecular glue degraders, on the other hand, has mostly come about by serendipity from phenotypic screening or through specific, well-characterized E3 ligase-targeting ligands (e.g., Cereblon). The researchers sought to identify a “chemical handle” that modularly attaches to different targeting ligands and converts these ligands into molecular glue degraders for the target proteins. The authors identified a minimal covalent chemical group that can be attached to multiple protein-targeting ligands to induce proteasome-mediated degradation of the target protein. This in turn enables the target-based modular design of molecular glue degraders.

KRAS G12C inhibitors are employed in treating solid cancers, including non-small cell lung cancer and pancreatic cancer caused by KRAS mutations (144–146). Most of these inhibitors target the inactive form of KRAS G12C, bound to guanosine diphosphate (GDP), which exhibits slow onset and rapid resistance (147, 148).Researchers shifted their focus to the active state of KRAS G12C bound to guanosine triphosphate (GTP). Taking inspiration from the mechanism of action of molecular glues (such as cyclosporine A, rapamycin, and FK506, among other natural products) that alter protein conformation by binding to receptors (such as the affinity protein FKBP12) and forming ternary complexes with target proteins (such as calcineurin), the CYPA:compound-1:KRAS G12C ternary complex was synthesized (149). Various structural optimizations were applied to this ligand, including indole substitutions and removal of the phenol hydroxyl group, compound RMC-4998 was produced (149). Individually, neither RMC-4998 nor CYPA could bind to KRAS G12C. However, RMC-4998 could alter its conformation by initially binding to CYPA, forming a CYPA: RMC-4998 dimer, and then covalently binding to the active state of KRAS G12C (KRAS G12C - GTP) to create a stable ternary complex (149). This ternary complex is inherently stable and exhibits excellent selectivity, binding neither to the inactive state of KRAS G12C (KRAS G12C - GDP) nor affecting wild-type KRAS, NRAS, or HRAS. The formation of the CYPA: RMC-4998:KRAS G12C ternary complex can inhibit ERK downstream signaling in KRAS G12C mutant cancer cell models, attenuate signaling in the AKT-MTOR and RAL pathways, and induce tumor cell apoptosis, demonstrating significant tumor inhibitory effects (149). It is currently in the clinical trial phase (NCT05379985), changing the situation where KRAS is an undruggable target, and specifically target the active status of KRAS G12C to achieve faster and more precise anti-tumor effects.

### 4.3 Deuteration

Deuterated drugs are a class of drugs in which the hydrogen atoms at specific positions in the molecular structure of the original drug are replaced by isotope deuterium atoms (150), and the main purpose of such drug reconfiguration is to optimize the pharmacokinetic (PK) characteristics and/or metabolic profiles of the original drug while keeping the original drug’s activity unchanged (151–153). The introduction of deuteration technology into glue design can stabilize its active conformation, enhance its activity, reduce side effects, and improve pharmacokinetic properties.

Avadomide monotherapy demonstrated acceptable safety and favorable pharmacokinetics in patients with solid tumors, NHL, and multiple myeloma (154). Researchers employed deuteration reactions to control the molecular chirality, yielding the biologically more active S-configuration of avadomide, known as DRX-164 (155, 156). Both DRX-164 and CRBN exhibit superior binding properties compared to the parent compound CC-122 (Kd 110 nM vs. 330 nM). Preclinical data illustrate that DRX-164 could induce apoptosis in multiple myeloma cell lines (151). In animal models, DRX-164 demonstrates enhanced therapeutic efficacy compared to lenalidomide and pomalidomide (151).

### 4.4 Template-assisted covalent modification

Based on the idea that covalent strategies could, in principle, facilitate molecular glue discovery by stabilizing new protein interfaces, Professor Nathanael S. Gray introduced an innovative strategy in the development of molecular glue degraders: Template-assisted covalent modification. In this approach, they engineered small molecules with modified charge groups to interact with BRD4 (157). Through the interaction between BRD4 and the DCAF16 protein, these charge-modified molecules prompted a reaction between their charge groups and a cysteine residue on the E3 ligase DCAF16 (157–159). This interaction induced the ubiquitination of BRD4, leading to its subsequent degradation.

BRD4, a vital epigenetic regulatory protein, modulates fundamental physiological processes like the cell cycle, proliferation, and apoptosis (160–162). Its pivotal role extends to tumor cell infiltration, metastasis, and tumorigenesis in hematologic malignancies such as AML, MM, and solid tumors like breast cancer, glioblastoma, and renal cell carcinoma (163–166). Leveraging “template-assisted covalent modification” to alter electrophilic covalent groups and directly modify the E3 ligase presents a promising strategy specifically for BRD4 degradation, offering potential as an anti-cancer therapeutic method.

## 5 Conclusions and perspectives

Remarkable strides have been made in the domain of targeted protein degradation, particularly in the development of molecular glue degraders, which have evolved from serendipitous discoveries to a more methodical and tailored approach in drug design (15, 140, 167, 168). Key innovations highlighted include the creation of chemical handles to enhance degrader activity, the fusion of natural molecular glues with drug ligands to impart pharmaceutical properties, and the use of isotopic labeling to optimize pharmacokinetics (140, 169–171). These advancements have been pivotal in the discovery of new therapeutic agents, opening fresh possibilities for targeting disease proteins that were once considered “undruggable”. Nevertheless, challenges remain in refining the PK and PD properties of molecular glue degraders. The incomplete understanding of PK/PD profiles for many preclinical candidates continues to hinder their clinical translation (172, 173). Future research must focus on identifying precise binding sites on target proteins, developing more specific linkers to enhance binding affinity, and improving the degradation efficiency of pathogenic proteins. These issues are particularly pressing in the context of cancer therapeutics. Looking ahead, exploring PPI and designing functional ternary complexes will be pivotal for advancing high-throughput degrader design (3, 174). We anticipate that more efficient screening methods—potentially based on substrate structure—will enable the identification and rational design of molecular glue degraders capable of targeting a broader spectrum of “undruggable” disease proteins. While substantial progress has been made in understanding the mechanisms underlying molecular glue action, significant gaps remain. In particular, a deeper understanding of the precise molecular processes involved, along with the development of more potent and selective inhibitors, is crucial for minimizing off-target effects. Furthermore, long-term safety and efficacy must be carefully evaluated, especially as these therapies advance into clinical applications. Overcoming these challenges will be essential to unlocking the full therapeutic potential of molecular glue degraders, and addressing these unresolved issues will provide critical guidance for their future research and development.

In conclusion, molecular glue degraders are emerging as a powerful and versatile class of therapeutics in tumor therapy. Their ability to target previously “undruggable” proteins, bypass resistance mechanisms, and selectively degrade oncogenic proteins presents a novel and promising strategy in cancer treatment. As research progresses, these agents hold significant potential to transform the landscape of cancer therapy, offering new avenues for treating both solid and hematological tumors.
